# A qualitative exploration of trial-related terminology in a study involving Deaf British Sign Language users

**DOI:** 10.1186/s13063-016-1349-6

**Published:** 2016-04-27

**Authors:** Alys Young, Rosemary Oram, Claire Dodds, Catherine Nassimi-Green, Rachel Belk, Katherine Rogers, Linda Davies, Karina Lovell

**Affiliations:** SORD (Social Research with Deaf People), School of Nursing, Midwifery and Social Work, University of Manchester, Jean McFarlane Building, Oxford Road, Manchester, M13 9PL UK; Institute of Population Health, University of Manchester, Jean McFarlane Building, Oxford Road, Manchester, M13 9PL UK; Mental Health Research Group, School of Nursing, Midwifery and Social Work, University of Manchester, Jean McFarlane Building, Oxford Road, Manchester, M13 9PL UK

**Keywords:** BSL, Sign language, Deaf, Trial terminology, Translation, RCT

## Abstract

**Background:**

Internationally, few clinical trials have involved Deaf people who use a signed language and none have involved BSL (British Sign Language) users. Appropriate terminology in BSL for key concepts in clinical trials that are relevant to recruitment and participant information materials, to support informed consent, do not exist. Barriers to conceptual understanding of trial participation and sources of misunderstanding relevant to the Deaf community are undocumented.

**Methods:**

A qualitative, community participatory exploration of trial terminology including conceptual understanding of ‘randomisation’, ‘trial’, ‘informed choice’ and ‘consent’ was facilitated in BSL involving 19 participants in five focus groups. Data were video-recorded and analysed in source language (BSL) using a phenomenological approach.

**Results and discussion:**

Six necessary conditions for developing trial information to support comprehension were identified. These included: developing appropriate expressions and terminology from a community basis, rather than testing out previously derived translations from a different language; paying attention to language-specific features which support best means of expression (in the case of BSL expectations of specificity, verb directionality, handshape); bilingual influences on comprehension; deliberate orientation of information to avoid misunderstanding not just to promote accessibility; sensitivity to barriers to discussion about intelligibility of information that are cultural and social in origin, rather than linguistic; the importance of using contemporary language-in-use, rather than jargon-free or plain language, to support meaningful understanding.

**Conclusions:**

The study reinforces the ethical imperative to ensure trial participants who are Deaf are provided with optimum resources to understand the implications of participation and to make an informed choice. Results are relevant to the development of trial information in other signed languages as well as in spoken/written languages when participants’ language use is different from the dominant language of the country.

## Background

Signed languages such as BSL (British Sign Language) are naturally occurring, grammatically complete languages [1]. They are not visual versions of the dominant spoken language of the countries where they are used. For Deaf people who use their native signed language, its use is a marker of cultural identity and a gateway to a common community whose traditions and histories in each country around the world stretch back over many centuries [2–4]. Yet Deaf communities around the world have experienced oppression through a failure to recognise their language and culture [2]. BSL only gained formal recognition as an indigenous language of the UK in 2003 [5] and only in Scotland has a law recently been passed conferring legal rights and duties towards BSL users [6].

English predominates as the language of education for Deaf children; for example, in England 87 % of deaf children only use spoken language in the school environment [7]. Fewer than 5 % of deaf children have one or more parent who is Deaf [8]; therefore, it is more usual for BSL *not* to be acquired from within one’s birth family. Also, Deaf culture is rarely passed inter-generationally with many young Deaf people forming their Deaf cultural identity as young adults through peers and Deaf community involvement [9]. In previous generations, it was more usual for deaf children to attend specialist deaf schools, often residentially, where sign language and Deaf culture were absorbed at an early age, even in those which were spoken-language educational environments.

In the United Kingdom, population estimates for BSL users range from 28,000 to over 100,000 [10] with a conservative estimate of around 70,000 being commonly cited [11]. Culturally, Deaf people who sign are often marked in literature by the use of a capital ‘D’ to distinguish them from the much larger population of deaf people who do not sign; a convention we follow here [12].

Although the health inequalities that Deaf people experience are increasingly recognised [13–16] and the distinct needs of Deaf people in terms of access to health services and care are a focus of interest [17], Deaf people are largely invisible in the clinical trials literature. The invisibility occurs both because Deaf sign language users are commonly excluded from trial participation because of the likely confounding variables they would introduce (see [18], chapter 5) and because there is almost no clinical trial work on an international basis that is focused specifically on Deaf people; for a rare exception see [19].

Using language to explore language, and in particular, terminology, has inherent challenges because language is both the subject and process of the endeavour; it is the referent and the means of reference ([20], p.15). Furthermore, familiarity with a word (lexical item) does not always confer familiarity with its meaning. In everyday life we all have knowledge of words whose meaning eludes us or words whose meaning we are sure of, only to realise that we have misunderstood. This is further complicated in communication between researchers of different disciplines, who attach different technical meanings to the same term. Communication between researchers and non-researchers faces similar problems compounded by a wide range of lay meanings attached to concepts and words. For example, ‘efficiency’ to the economist means maximising benefit for a given budget or minimising the cost of achieving a given outcome. In lay terms, for the general population and other research disciplines, ‘efficiency’ is often used to denote simply cost-cutting with no regard to outcome.

Yet using language to talk about language and its meaning is not an impossible task. Terminology (‘jargon’) can act like a placeholder – its potential ambiguity, obscurity, lack of transparency or multiple meanings are accepted on a temporary basis whilst it is talked *about*. Encyclopaedic understanding of how the words we use are defined is usually not required for us to use them in everyday life [21]. We can choose our words on the basis of our linguistic knowledge (ibid) only, having experience of when or how terms might be used without fully understanding them.

This approach has formed the basis of many studies which have sought to understand participants’ understandings of familiar terminology in clinical trial designs and the common assumptions associated with words such as ‘randomisation’, ‘trial’, ‘consent’, ‘placebo’, ‘arm’[22–24]. The findings have revealed understandings and misunderstandings of key concepts and have been used to help improve participation in trials through ensuring that the information is relevant, appropriate and accessible [25, 26]. From an ethical perspective, supporting understanding and avoiding misunderstanding are crucial to informed consent.

In relation to Deaf participants who use BSL, three key sources of complexity in producing relevant and accessible recruitment-related information are likely to be: (1) background (general) knowledge considerations associated with Deaf people, (2) properties of a visual spatial language, and (3) bilingual considerations.

In terms of background knowledge considerations, Deaf people routinely experience considerable barriers to accessing information and the acquisition of knowledge, whether deliberately or incidentally. In part, this is because Deaf people commonly experience highly limited access to information on a wide range of everyday subjects because it is not available in a signed language [27, 28]. Secondly, the majority of Deaf people who have been deaf since birth or early childhood, have lower than average levels of literacy in the written word in comparison with hearing people [29]. Also, the acquisition of incidental and everyday information is hampered by limited access to the spoken word. Finally, a paucity of peers who sign in the general environment reduces further the opportunities to pick up knowledge through casual conversation. Consequently, it is recognised that many Deaf people experience what has been termed a ‘low fund of information’ [30]. Therefore, many Deaf people, who are potential trial participants, might not be even casually familiar with the terms and concepts used in participant information sheets, informed consent forms and the verbal support available from researchers.

In relation to the properties of a visual, spatial language, there are features of a signed language that potentially raise different challenges to spoken languages, which use terminology as temporary placeholders to explore understanding. For example, ambiguity and non-specificity can be harder to convey in a language where visual specificity is integral to those signs that are iconic in nature [31]. A phrase such as ‘kill oneself’, for example, that in English remains without any indication of means, in BSL would be more difficult to convey without an assumption of manner of death in how it is signed (see [32] for more examples).

Signed expressions that operate as temporary placeholders to facilitate discussion are also potentially problematic within a visual language. This is because their form can influence the conceptual understanding of participants unfamiliar with the term; the shape, orientation or movement involved in a signed expression can reveal underlying assumptions associated with its meaning. For example, EXPERIMENTAL STUDY^1^ might be expressed, at least initially, using signs drawn from the common lexicon, such as EXPERIMENT followed by STUDY, until the true meaning can be discussed and an appropriate signed phrase arrived at. However, the *sign* for EXPERIMENT is not vague; it is usually an iconic representation of test tubes being poured. This immediately introduces the notion of laboratory science rather than an experimental study potentially involving complex interventions of people, behaviour and therapies. The temporary placeholding sign sets up unwelcome initial assumptions and implications because of *how it looks* even if those involved are aware it is acting as a placeholder. Finger spelling^2^ the English form of a term is a common way to get round this problem – one may spell out e-x-p-e-r-i-m-e-n-t-a-l – however, to do so introduces a bilingual aspect into an otherwise monolingual discussion and requires acts of translation.

In terms of bilingual considerations, although the first and/or preferred language of Deaf trial participants is BSL, no Deaf person in England is growing up in a monolingual environment. The written word and the spoken word are everywhere. Deaf individuals will, therefore, have differing degrees of access to, and familiarity with, English (or other spoken/written languages) that will have an influence on conceptual understanding as well as basic knowledge. There are examples where the written form of a word might influence the signed lexicon, e.g. the Isle of Wight used to commonly be signed as ISLE of WEIGHT. In other cases the phonetic properties of the surrounding spoken language can be an influence as in the case of Preston (the city), being signed as PRIEST, because the shape on the lips of Preston and Priest are similar. Also for Deaf people who, like hearing people, may be prodigiously bilingual or multi-lingual, there are just some words and expressions more familiar in one language than another and the ability to code switch between languages can be a strength.

In 2014 we were funded by the National Institute for Health Research in England to undertake the preparatory work required for a feasibility study leading to a clinical trial involving Deaf people in the context of a primary mental health intervention (http://www.nets.nihr.ac.uk/projects/hsdr/1213679). The programme known as BSL Healthy Minds [33] is an adaptation of the National Institute for Health and Care Excellence (NICE) approved psychological intervention: Improving Access to Psychological Therapies (IAPT) [34]. It is delivered in BSL by Deaf trained practitioners and aimed at Deaf people experiencing common mental health disorders including depression and anxiety. In line with the Medical Research Council (MRC) framework for complex interventions [35] a range of preparatory studies were undertaken, one of which is the focus of this paper: namely, exploring lay understanding amongst Deaf people of key terminology and concepts associated with clinical trials. This was a necessary precursor to creating linguistically and culturally appropriate recruitment, information and consent materials for the forthcoming clinical trial.

However, there were no previous randomised controlled trials (RCTs) that involved Deaf people who use BSL. This meant that we were not seeking to improve participation but rather to facilitate participation in the first place. It also meant that there was no confirmed lexicon in BSL for common terms associated with clinical trials. This was true both of the sign bilingual university research department carrying out the study and for the everyday contexts of Deaf lives and conversation. Vocabulary and relevant forms of expression in any language only emerge when a population has direct experience of a topic; think, for example, of the evolution in English of vocabulary to match the explosion in information and communication technologies. As a consequence, the focus of our work in the study reported here was *not* on the translation into BSL of key terminology associated with clinical trials; it was on its conceptual exploration with a linguistic community in order to identify best means of signed expression and explanation, and likely barriers to comprehension.

Specifically our aims were to:

- Explore, in BSL, the meaning and understanding of key concepts and common lexical items associated with recruitment and consent to a clinical trial
- Enable signs/signed expressions to emerge that are semantically accurate and support Deaf people’s informed consent in any future trial
- Identify key points that can inform the continued development of acceptable and accessible participant information for Deaf people who use BSL and are recruited to clinical trials

We anticipated that many participants in our study might not be even casually familiar with the terms and concepts we would be exploring, and that access to information that might support understanding of unfamiliar terms would be more limited than amongst hearing lay communities.

## Methods

### Methodological approach

The qualitative research design is underpinned by a phenomenological approach [36]. Phenomenology draws attention to sense-making through social and communicative interactions and emphasises that meanings are not fixed but are generated in context through the use of language and the prior experiences and culture that individuals bring to an interaction [37]. This was an appropriate approach for a study that was exploring conceptual understanding of terms through group-based discussion (focus groups). It is also an approach that enables cultural meanings to emerge through analysis rather than a priori definitions to be assumed.

### Recruitment

An explanation of the study in BSL was posted on the research group website and advertisements to participate in the focus groups were placed on a signed Facebook site accessed by the Deaf community and through email, shared networks and word of mouth/word of hand. A purposive sample was sought of Deaf people aged 18 years or over, who used BSL as their first or preferred language. Anyone currently receiving support through the IAPT programme was excluded because additional ethical permissions, over and above those required for an exploratory study, would be required if participants included those who were current patients of the National Health Service in England.

### Ethics

The study was approved by the University of Manchester Research Ethics Committee (Ref: 14183). Informed consent was obtained from all participants. Participant information sheets were made available in advance of the focus groups in BSL on a website as well as in plain English. Prior to the focus groups, the researchers clarified the information in BSL again, face to face, and participants had the opportunity to ask questions. All participants were provided with a pre-written, postage paid withdrawal form to facilitate easily withdrawal of consent for their data to be used subsequent to the focus groups.

### Data capture methods

Data were collected through focus groups with Deaf people which were facilitated in BSL by researchers who are native signers (RO and CNG). Four focus groups were held, in three different regions in England, involving 19 people in total (one group chose to meet on two occasions rather than once for a longer period of time). The groups were in two parts, each lasting between 1.5 and 2 hours with refreshments provided. In part 1, participants were introduced to the purpose of the study and clinical trials in general. In part 2, discussion focused more specifically on how to provide good information in BSL to support recruitment and informed consent. The specific terms that the group were asked to discuss were: ‘randomisation’, ‘feasibility’, ‘informed choice’, ‘trial’, ‘consent’ and ‘experimental study’. They were informed in advance that this would form the content of the discussion. There were no interpreters present at the focus groups because the facilitators, RO and CNG, are native BSL users sharing a common language with participants. Three cameras were used to capture the discussions. These were time-coded enabling the later simultaneous display of all interactions and communication for purposes of analysis. PowerPoint was initially used as a prompt to different sections of the discussion. In some instances the prompts were visual diagrams, e.g. showing two arms of a trial and how it relates to initial recruitment of a sample; in others the prompts were specific words written in English that could be referred back to as prompts during the discussion.

### Participants

All participants were over the age of 30 years with three over the age of 61. Of the 19, 2 were unemployed and 4 retired. The rest were in employment although the majority of these were in part-time employment. The entire sample had a self-declared strong Deaf identity. Table 1 shows distribution of numbers per group and characteristics.

**Table 1:** Participant characteristics

Groups	Participants	Gender	Ethnicity	Deaf parents?	Age BSL acquired	Involvement in Deaf community	I feel I am culturally Deaf	Highest qualification
Group 1	1a	Male	White British	Yes	From birth	Often involved	Very much so	Postgraduate diploma
	1b	Male	White British	No	4–7 yrs	Often involved	Very much so	Vocational qualification
	1c	Female	White British	No	12–16 yrs	Often involved	Quite so	School leaving certificate
	1d	Female	Asian Indian British	No	Over 25	Very involved	Very much so	Vocational qualification
	1e	Female	White British	No	Missing	Often involved	Quite so	Professional diploma
Group 2	2a	Female	White British	No	1–3 yrs	Missing	Very much so	On-the-job training
	2b	Female	White British	No	4–7 yrs	Very involved	Very much so	Vocational qualification
	2c	Female	White British	No	4–7 yrs	Very involved	Quite so	Missing
	2d	Female	White British	No	4–7 yrs	Started age 40	Very much so	Vocational qualification
	2e	Female	White British	Yes	From birth	Often involved	Very much so	Missing
Group 3	3a	Male	Asian Indian British	Yes	Over 25	Often involved	Quite so	Missing
	3b	Female	White British	No	1–3 yrs	Often involved	Very much so	Professional diploma
	3c	Male	White British	No	From birth	Often involved	Quite so	Professional diploma
	3d	Female	White British	No	8–11 yrs	Often involved	Very much so	Postgraduate certificate
	3e	Female	White Jewish	No	Over 25	Often involved	Somewhat	Professional diploma
Group 4	4a	Female	White British	No	4–7 yrs	Very involved	Very much so	University degree
	4b	Female	Jewish	No	17–24 yrs	Often involved	Somewhat	Missing
	4c	Female	White British	No	17–24 yrs	Very involved	Very much so	Postgraduate certificate
	4d	Female	White British	No	Over 25	Often involved	Quite so	Professional diploma

*BSL* British Sign Language

### Data analysis

All data were kept in their source language for purposes of analysis. The video files were uploaded to Nvivo 10 which has the facility to tag and segment video data, in this case visual language data, for purposes of thematic coding without the need to transcribe data. This was important because if the data were transcribed this would equal translation in the case of BSL data which have no written form. This contrasts with many spoken languages where to transcribe data is only to change its modality (from spoken to written) and not to translate [18, 38–40]. All data were watched and re-watched independently by two researchers (CD and CNG) with the aim of creating an initial coding framework.

The two researchers carrying out the analysis brought different personal and professional biographies to the task: one is a native Deaf sign language user from a Deaf family who has worked for over 10 years in research roles; the other is a hearing researcher who learned BSL as an adult and who has been a qualified and registered sign language interpreter for 11 years, in addition to her research role. Their initial coding frameworks were compared and discussed with a third researcher (AY) who was overseeing the analysis (a hearing late learner signer who has worked in the Deaf studies field for 25 years). Many of the same themes had been identified arising from specific examples in the data but were not clustered in exactly the same way in how they had been organised by the two researchers. Further, discussion led to a framework consisting of the following four areas under which there were additional layers/sub-themes (Table 2).

**Table 2:** Structure of data analysis, themes and sub-themes

Theme	Sub-themes
Strengths and challenges arising from properties of a visual language	Acceptance of generality/specificity
	Verb directionality
Conceptual understandings/misunderstandings of common terms	Orientation toward avoidance of misunderstanding
	Substitution of alternative words/expression
Bilingualness and English influences on understanding and expression	Visual decoding of English words
	Tests not discussion
Power differentials in acquiring and generating new knowledge	Perceptions of class
	Language in use

## Results and discussion

The following presents *illustrative examples* for each theme/sub-theme rather than an exhaustive description, for there were myriad examples associated with each that emerged from the analysis.

### Strengths and challenges arising from the properties of a visual language

#### Acceptance of generality/specificity

BSL is a four-dimensional language, encompassing the usual spatial dimensions and time [41]. Users combine signs from the established lexicon with productive signs (i.e. those produced in the moment to match intended expression), or classifier proforms, a term which in sign languages refers to a handshape used to describe the appearance, location, and/or movements of objects. This gives the utterance a high degree of specificity, something we would not necessarily expect to see in a corresponding English phrase. For example, whilst in English we might say ‘she opened the window’, in BSL the corresponding signed phrase would include specific details of the form and movement of the window being talked about; was it a sash window, a window that opened from the left, or the right, or a tilt window? The correct form and movement of the window would be reproduced in how the phrase ‘she opened the window’ was signed.

In the focus groups, there were numerous examples of how this *accustomed expectation of specificity* was both helpful and unhelpful to participants exploring the meaning of unfamiliar terms; for example, RANDOMISATION. One approach to signing this conceptually would be to set up in space two locations, one to the right and one to the left, to which people might be randomised as in differing arms of a trial. Given that in BSL space carries semantic significance, this approach clarifies easily the notion of there being two potential destination groups to which one might be randomised. However, in one of the focus groups, the signing of randomisation to incorporate two destination spatial locations led to the not unreasonable question of whether there was ever the possibility of randomisation into more than two groups? If so, then this element of how randomisation might be signed would have to be modified to encompass three or more visual locations in front of the signer to be accurate. Expectations of visual specificity were being linked to the methodological basis of the trial design; thus, warning of the importance of accuracy at this level if later misunderstanding were to be avoided.

In another group, participants felt strongly that it was important to know where the things/people to be randomised were being drawn from; in one case, it was suggested the sign for RANDOMISATION be preceded by an indication of the whole of England to make explicit the idea that participants could come from across the country. This in turn would modify how the idea of randomisation would be expressed. Of course, written information sheets in English, for example, might include information about the scope of recruitment under a section on why the individual is being asked to participate. However, in this example, the difference is that participants were of the view that *where* people might be drawn from directly affected *how* the concept of randomisation should be expressed in BSL. This contextual, semantic layering of aspects of information within a single expression is very common in signed languages, whereas in written languages a sequential approach to aspects of information is more usual.

Another aspect of BSL, handshape, also created potential for both confusion and illumination with respect to understanding RANDOMISATION because of high expectations of specificity. In BSL, handshapes may iconically take on the visual representation of a subject/object as in the commonly used shape for a telephone, for example, which involves extending the thumb and little finger whilst folding away the others to make a representation of a telephone receiver or handset. Handshape in BSL can also take on a classifier form, that is to say conventionalised (stylised) shapes that stand in for a subject/object, such as a flat hand for a car, or an extended vertical index finger for a person. In our study, one of the potential ways to sign randomisation involved movement away from the body by each of the hands sequentially toward the destination locations whilst shaping each hand to represent what it was that was being randomised. By utilising a handshape that is the classifier for an individual or the handshape that is the classifier for a group, the same signed expression could either indicate an RCT where the individual is the unit of randomisation or an RCT where the group is the unit of randomisation. But it is the same expression; the slight alternation of the handshape is what reveals this aspect of the underpinning research design.

In summary, BSL users often expect visual specificity to be present, and for it to be grammatically correct in relation to what is being described. This means they may be less tolerant of, or less comfortable with, generality within statements than English speakers may be. Consequently, it is important that the signs to describe and explain randomisation are both grammatically correct and conceptually more specific than the English written or spoken counterpart. This involves, essentially, paying close attention to the visual grammatical properties of signed languages and ensuring they accurately portray the underpinning trial design so as to promote conceptual understanding and avoid later misunderstandings by those participating. The need for visual specificity is a key step in developing conventionalised signs for participant information and recruitment into prospective randomised and non-randomised evaluations.

#### Verb directionality

Another feature of BSL which is not present in English is verb directionality. Where in English the directionality of the verb must be described separately from the verb itself (e.g. Sarah asked Jane), in BSL once Sarah and Jane have been established in signing space, the movement of the verb TO ASK from one place to another is sufficient to specify who is asking and who is being asked. This feature generated considerable discussion within the groups, particularly with reference to the idea of INFORMED CHOICE.

In English, ‘informed choice’ can be confusing given that ‘informed’ as an adjective implies having or showing knowledge of a subject or situation, and as a verb is the past tense of ‘inform’; to give (someone) facts or information. Thus, to be informed is to be knowledgeable *and* is to have been told by someone else. The potential ambiguity of the meaning of ‘informed choice’ nonetheless usually does not obscure the fact that the individual subject is making a choice. In BSL, however, the individual as the receiver of information on which to make a choice can be explicitly shown through the direction of the verb TO INFORM as it is signed; the information is literally shown as being received into one’s head, having been given by another.

Leaving aside the nicety that information might be ‘felt’ rather than ‘known’ [42] and, therefore, could be signed as entering the body at the level of the heart or stomach (gut feeling), it is the visual verb direction that clarifies any potential confusion. However, if signed in the opposite direction, information is being given away to another, rather than received. Yet this might not necessarily be incorrect because it implies choice is being given to the individual which after all is one of the purposes of good information. Indeed there was much discussion (and confusion) in the groups about the difference between informed choice implying I HAVE TOLD YOU (‘I have told you what my choice is’) rather than I HAVE BEEN TOLD (‘I have been told what my informed choice options are’). The general questioning of the *directionality* of the verb in how INFORMED CHOICE was being signed, in reality uncovered and distinguished all of these strands of what informed choice actually meant. It showed the complexity involved in signing this well to prompt all of these aspects of understanding. Verb directionality distinguished them explicitly rather than in the passive English form where they are implicit.

#### Discussion

In recent years, research in the wider field of information to support recruitment to clinical trials has placed emphasis on distinguishing between understanding and comprehension. Understanding concerns the core meaning of lexical items and can be referred to as ‘linguistic knowledge’ [21]; words are recognised and familiar. Comprehension refers to the additional aspect of grasping the meaning and implications of a specific term within the context in which it is used (semantic meaning) and sometimes referred to as ‘conceptual knowledge’ (ibid). Studies of language in trials have demonstrated, for example, that there is a difference between understanding the mechanics of a process, such as randomisation (how it occurs), and participants’ comprehension of the purpose of that process (why it is necessary and its implications) [23–25]. It is argued that both are required for informed consent and recall of one might hide misapprehension of the other.

In our study, this important distinction between understanding and comprehension is operating at a more fundamental level; the foundational vocabulary and underlying concepts in which to present and discuss involvement in a clinical trial are largely missing. In one sense this is perfectly normal. Many languages have yet to develop a lexicon in some domains if the users of that language are yet to have encountered the topic area; think of the inadequacies of English to discuss information and communication technologies until the Internet age forced the development of an appropriate lexicon.

By encouraging an exploratory, discursive approach to clinical trial terminology in BSL we were able to observe (literally) participants’ (mis)understandings because these were revealed in how the visual grammar of potential signed expressions were modified. By asking critical questions about *how* something is signed enabled a growth in comprehension *about* details such as the underlying methodological design. In turn, growing comprehension of what was intended by such terms as ‘informed choice’ and ‘randomisation’ facilitated a more critical awareness of whether the approach to its explanation in BSL was adequate or required modification. Understanding and comprehension were simultaneously addressed through a conscious exploration of the properties and resources of the visual language to arrive at the best expressions possible.

### Conceptual understandings and misunderstandings of common terms

#### Orientation toward avoidance of misunderstanding

One of the important functions of the focus groups was to arrive at, through open exploration such as that illustrated so far, some ideas from participants about how best to explain a concept so that it is understood well when expressed in BSL. It is interesting that many of the discussions in this respect centred on how to avoid misunderstandings, rather than how to best express an idea. This suggested that participants were very used to misunderstandings in situations of information exchange. For the majority, this leaning toward strategies to avoid misunderstanding is likely to have grown from being usually faced with information in English, rather than BSL and, therefore, trying to navigate meaning from the less familiar or fluent language, including relying on guesswork when lip-reading [17]. This orientation was retained despite being involved in a situation focused on best expression in first/preferred language. Perhaps it arose in part also from the presence, as prompts, of some terms in English alongside terms expressed in their placeholder form in BSL. Whatever the exact origin, it strongly influenced some of the groups’ suggestions.

For example, even when signed in BSL, group members reminded us that certain signed expressions will carry meanings derived from familiar contexts which influence any additional meaning in a new context. A key example of that was TRIAL. Participants associated the English word ‘trial’ with going to court, taking tablets for a trial period, a work trial that may result in a permanent job and the slime left behind by a snail (in reality ‘trail’ in English but it has the same lip-pattern as trial). Two of these contextual associations, taking tablets for a trial period and a work trial, when expressed in BSL would use a sign for TRIAL that could be used in the context of a clinical TRIAL. However, the other contextual associations demonstrate just how easily misunderstanding might arise particularly if all of the four examples above utilise the same lip-pattern as previously discussed. One group finally suggested that the lexical item for TRIAL should be dropped totally when used in the context of a clinical trial and instead a compound sign akin to HAVE A LOOK would be more appropriate. Yet none of this actually captures what is meant by a ‘clinical trial’.

#### Substitution of alternative words/expressions

Another example of a very fruitful discussion on avoiding misunderstandings concerned CONSENT. The most common signs for CONSENT are usually those implying AGREE or APPROVAL. Whilst these are aspects of consent, the discussion in one of the focus groups attempted to get to the bottom of what consent actually meant in the context of a research study when a potential participant is asked to give their consent. The result of the wide-ranging discussion was to suggest to the researchers that a compound sign (i.e. several linked signs standing for one word) and implying PERMISSION was a more accurate way to express this because a participant was giving their permission rather than agreeing with or approving of what a researcher may be telling them.

#### Discussion

Other studies in the mainstream have demonstrated that participants will apply their pre-existing knowledge of words in a familiar context to understand their meaning in the unfamiliar one (clinical trial) [22, 23]. This was evident in our data too but with the additional complication of bilingual decoding strategies that participants might use between languages to make sense of unfamiliar ideas and the visually perceived nature of English words that can lead to misunderstandings. In addition to our findings alerting us to some specific problematic linkages between terms (e.g. TRY/TRIAL), the findings reinforce the importance of understanding language in the context of its users; in this case Deaf users of BSL existing in a hearing/spoken/written dominant language world.

Contextualisation is a growing area of interest with respect to trial design and explanation of effect with respect to generalisation [43] but has received far less attention with respect to language use and specifically bilingual or multi-lingual trial participants’ engagement with trial information materials. Regardless of one’s dominant language, anyone who is bilingual or multi-lingual will experience influences on comprehension that derive from knowledge and use of languages other than that of the language with which they are engaging at the time. Particularly for potential participants in contexts where one language may be regarded as official or dominant but on an everyday basis people might use several others, the influence of bilingual or multi-lingual status is potentially significant as well as the differential status between languages in context.

In respect of our study, we have shown that the identity of Deaf people who might be dominant BSL users but who nonetheless contextually engage in everyday acts of translation and decoding of the majority language, does impact on comprehension of the unfamiliar. However, more specifically, the common experience, or rather expectation, of misunderstanding information deriving from everyday necessary encounters with the less fluent language (English) has also produced a strong orientation toward avoidance of misunderstanding. This requirement for any future information production was strongly emphasised by our participants. It was not just a product of a bilingual status but also of a social status whereby equivalence of access in both languages was not usually offered; thus, socio-linguistically producing an internalised preference for forms of explanation explicitly designed to avoid misunderstanding. For example, one could imagine any future information materials explicitly pointing out that ‘trial’ does not mean ‘try’ and giving an explanation of TRIAL with examples as a preface to any subsequent formal presentation of trial-specific information materials.

### Bilingualness and English influences on understanding and expression

#### Visual decoding

All Deaf BSL users are bilingual to some degree. They exist within an environment where exposure to written English is inevitable and, like any users of any language, they employ strategies and tactics to help them make sense of unfamiliar words or phrases. As Deaf BSL users generally have limited or incomplete auditory exposure to spoken English, the strategies employed are more likely to be *visual* in nature. For part of the discussion, key words in their English form were used as prompts for the discussion and our data illustrate a number of different strategies used by participants to attempt to unlock meaning based on visual recognition of words or parts of words.

For example, although participants were unfamiliar with the word ‘randomisation’ they recognised ‘random’ as a familiar word, and used their conceptual knowledge of that term as a basis for discussion. This led to some useful exploration of associated concepts like ‘chance’, and ‘without pattern’ which in turn supported comprehension of ‘randomisation’.

When discussing ‘feasibility’, a number of participants recognised the suffix ‘-ibility’. Again, they used the familiar part of the word to drive their discussion. In this instance, the tactic was far less successful, as the suffix was retained and the root of the word discarded and replaced with a variety of alternatives in an attempt to understand the term as a whole. Unrelated terms such as possibility, responsibility, flexibility, ability, variability, availability (of information), entered the discussion but were not helpful in illuminating conceptual understanding.

#### Tests not discussion

With hindsight, the reference in the groups to key terms in English, even though secondary to explanation and exploration in BSL, proved problematic. The problem did not derive from the presence of English terms per se. As discussed previously, no Deaf person is truly monolingual and most will navigate in everyday life through the maze of English to different extents. The problem lay in what the use of unfamiliar words in English, regardless of the discussion being in BSL, actually *represented* for many participants.

As previously discussed, the vast majority of Deaf people are educated in spoken/written English with late acquisition of BSL being more common. Many Deaf adults recall struggles to learn in English through spoken and written language and some retain painful memories of what they regard as an education system that has withheld their right to be educated in a signed language [4]. Given this context and the age of the participants, in our study there was evidence of some of the participants interpreting the focus group discussions as a form of test, whether of word recognition in English or conceptual understanding in BSL. This was manifest in repeated phrases like ‘I may be wrong but…’ and the body language of some participants who, in the midst of exploration in their strongest and preferred language, nonetheless interpreted the discussion as threatening and revealing of them ‘not knowing’. We would suggest that this response may be linked to negative memories of school-age education where they might have struggled for access to knowledge. Consequently, open discussion can feel more like a test than an ideas exchange and participation interpreted negatively as revealing of ignorance rather than contributory to new knowledge.

### Power differentials in acquiring and generating of new knowledge

#### Perceptions of class

The focus groups were set up to maximise the commonalities of language and culture between the researchers and participants. This might seem obvious but it is far more common internationally for hearing people to carry out research with, or in some cases on, the Deaf community and usually using interpreters, particularly in focus groups (see, for example [44]). Deaf-to-Deaf exchanges like the one described in this study remain the exception, not the rule. In collecting data in this way we had felt confident it would be a successful approach to elicitation and exploration. However, the discussion groups consistently revealed awareness amongst participants of difference and also deference.

There were numerous references amongst participants to ‘clever Deaf’ (implying the group facilitators) in contrast to self-referential phrases such as ‘ordinary Deaf’ or ‘Deaf like us’. Even though both researchers were well-known people in the Deaf community and, furthermore, from Deaf families, this did not alter the sense of separation that was expressed as well as the sense of commonality.

#### Language in use

In many respects perceptions such as these should not be a surprise as divisions of class, intellect and education are common across all communities [2]. However, it was a timely reminder, in terms of producing information materials for any future clinical trial, that accessibility for Deaf people is not defined by simply ensuring the material is in BSL, it should be in the BSL that is recognisable to the common Deaf population, not to those who are the intellectual and academic elite in the Deaf population.

#### Discussion

With respect to written languages, guidelines governing acceptable reading age for public information materials do not actually address what the *language-in-use* currently is within any given community. A term might be in plain English, but it may not be what everyone actually uses. Investigation of terms and expressions in use, therefore, is also an important aspect of generating materials that support comprehension. It is an approach that is increasingly informing a wide range of research and educational endeavours aimed at making a difference within specific socio-cultural communities (see, for example [45]). In relation to this project, there were no current terms in use for the topics we were exploring, but the act of doing so began the process of the community generating its own. The job of the researchers, therefore, will be to capture those and use them in any future clinical trial information, perhaps through online vlogs in signed languages, as the community begins its own conversations about participation in clinical trials.

## Conclusions

### Key recommendations

Maximising the comprehension of participant information materials for recruitment to clinical trials is a significant ethical requirement. We have identified six necessary conditions that need to be met when developing signed participant information for Deaf people so that it is acceptable, accessible, transmitted accurately and understood as intended. They are required to address the cultural preferences and lower background knowledge of Deaf people. These are likely to apply to all signed languages, not just BSL. All of them also potentially apply to further development of written and spoken information for hearing participants for whom the majority language (English) is not their first or preferred language:

1. A community-participatory, exploratory approach to arriving at appropriate clinical trial terminology is highly effective in instances where languages, in this case BSL, have not yet had the contact with a topic that would mean a common vocabulary/preferred means of expression has developed
2. Languages have properties associated with their form and grammar that naturally enable some approaches to explanation to support comprehension that others may not. In this case, verb directionality, expectations of specificity and simultaneous contextual, semantic layering within expressions enabled features of the underpinning trial design to be clarified and remain consistent
3. It is important to take into consideration bilingual influences on comprehension even when information is presented monolingually; this is a decoding strategy for unfamiliar terms and concepts that is available to those who are bilingual and multi-lingual and can be a source of both strength and misunderstanding
4. Orientation of information to avoid misunderstanding is an important axis to consider when creating new information for a cultural-linguistic group unfamiliar with the topic. It is subtly different from an orientation designed to support comprehension and may, as in the case of the sample in this study, be a preferred orientation
5. The researcher should understand cultural, contextual or social barriers that participants might face in engaging in open, constructive discussions of the information materials and consent procedures, over and above those that might be created by language per se. In the case of Deaf people, these barriers might derive from negative historical experiences of the education system, and class differentials
6. Clarity of expression, in the sense of plain language or avoidance of jargon, is not sufficient to promote comprehension. Attention to language-in-use in contemporary discussion is an important means of expression to effectively communicate complex concepts because it reflects common cultural usage. Simple, straightforward language may still seem alien if it not recognisable as something shared and used within a given community

### Next steps for research

These findings represent only the first step in exploring holistically the best conditions for engagement with clinical trial information for users of a signed, visual language. It stops short of two additional considerations which will be addressed in later stages of this work. The first concerns engagement with information materials and the influence of forms of presentation. There is a plethora of evidence in the mainstream that interaction between recruiter and participant over the information provided leads to greater comprehension, participatory decision-making and more informed consent than passive reading of materials alone [24, 26]. For a visual language with no written form, testing out the additional benefits for comprehension and informed consent of dialogue between recruiter and potential participant, whilst jointly viewing the video of the information materials versus the participant viewing the information alone, would be worthwhile.

Second, the form of presentation of the materials in BSL requires further investigation. Deaf communities around the world are well-known for preferring visual materials, such as diagrams and pictures, in addition to signed explanations because of visual cognitive strengths and preferred visual-thinking strategies [46]. Therefore, how materials in signed languages are presented is also crucial. Previous studies have explored layouts on computer screens and the role of options to view in the dominant written language of the country as well as its signed language [47]. The scope for active dialogic presentation of information sheets in a signed language, forming a conversation to be viewed rather than a passive watching of materials, is another potential avenue for development [19].

### Limitations

The participants in this study may not be representative of the diversity of Deaf users of BSL. They engaged with new ideas and concepts on only two linked occasions and, with greater consideration and time, may have had more to contribute to the development of the study.

## Abbreviations

BSL: British Sign Language
IAPT: Increasing Access to Psychological Therapies

## Footnotes

1 Capitalisation of words is an accepted convention to imply that the signed utterance is being referred to.

2 Fingerspelling refers to specific and conventionalised hand movements standing for the English alphabet, A, B, C, etc. that can be combined to spell out, letter by letter, the English word or name being referred to.
